# Characterization of a dual function macrocyclase enables design and use of efficient macrocyclization substrates

**DOI:** 10.1038/s41467-017-00862-4

**Published:** 2017-10-19

**Authors:** Clarissa M. Czekster, Hannes Ludewig, Stephen A. McMahon, James H. Naismith

**Affiliations:** 1 Biomedical Sciences Research Complex, The University of St Andrews, North Haugh, St Andrews, K16 9ST UK; 2 Biotherapy Centre, Sichuan University, Chengdu, China; 3 RCaH, Rutherford Appleton Laboratory, Harwell Oxford, Didcot, OX11 0FA UK; 4 Division of Structural Biology, University of Oxford, Henry Wellcome Building for Genomic Medicine, Old Road Campus, Roosevelt Drive, Headington, Oxford, OX3 7BN UK

## Abstract

Peptide macrocycles are promising therapeutic molecules because they are protease resistant, structurally rigid, membrane permeable, and capable of modulating protein–protein interactions. Here, we report the characterization of the dual function macrocyclase-peptidase enzyme involved in the biosynthesis of the highly toxic amanitin toxin family of macrocycles. The enzyme first removes 10 residues from the N-terminus of a 35-residue substrate. Conformational trapping of the 25 amino-acid peptide forces the enzyme to release this intermediate rather than proceed to macrocyclization. The enzyme rebinds the 25 amino-acid peptide in a different conformation and catalyzes macrocyclization of the N-terminal eight residues. Structures of the enzyme bound to both substrates and biophysical analysis characterize the different binding modes rationalizing the mechanism. Using these insights simpler substrates with only five C-terminal residues were designed, allowing the enzyme to be more effectively exploited in biotechnology.

## Abstract (web summary)

Cyclic peptide macrocycles are promising anti-cancer and antimicrobial molecules. Here, the authors characterize the structure and catalytic mechanism of the prolyl oligopeptidase B from Basidiomycete fungi, showing that its dual macrocyclase-peptidase activity is crucial for amatoxin macrocyclization.

## Introduction

Cyclic peptide macrocycles hold promise in pursuing challenging targets involved in protein–protein interactions implicated in diseases as diverse as cancer and antimicrobial infections^1^. Due to their constrained, pre-organized, and protease-resistant structures, these molecules can modulate key complex macromolecular interactions in a manner that has proven extremely difficult for conventional small molecules^1, 2^. In contrast to most linear peptides, many peptide macrocycles are highly cell permeable^3^. Ribosomally synthesized and post-translationally modified peptides (RiPPs) are a particularly attractive class of macrocycles because their enzymatic synthesis is driven by enzymes working in cascade to process a genetically encoded and highly variable peptide precursor^4^. The peptide precursor can be modified by macrocyclization, oxidation, heterocyclization, hydroxylation, and prenylation in a predictable and scalable manner^5^. The patellamide pathway is a paradigm in this system, in which catalysis and recognition are physically separated in many of the enzymatic steps leading to a unique combination of specificity and promiscuity^6^. The macrocyclase in the patellamide biosynthetic pathway (PatGmac) belongs to the subtilisin class of proteases, requiring a substrate with a C-terminal AYD motif, preceded by heterocyclized cysteine or a proline residue^7, 8^. The enzyme is otherwise almost insensitive to the core sequence that becomes the macrocycle and only the thiazoline (or proline) are part of the final product, as the AYD is cleaved off during the reaction. This combination of specificity through the use of disposable tags (leader and/or tail sequences) and promiscuity in the core sequence produces a system that is almost infinitely variable. This has made RiPPs appealing for exploitation in biotechnology. In some RiPPs systems, a linker that can also be varied in both length and composition separates the recognition tag and core peptide^9, 10^. Despite the appeal of their promiscuity, the PatG family of macrocyclases face a severe drawback as they are slow^11, 12^, although in vitro addition of reductant does increase catalytic efficiency^13^.

**Fig. 1:**
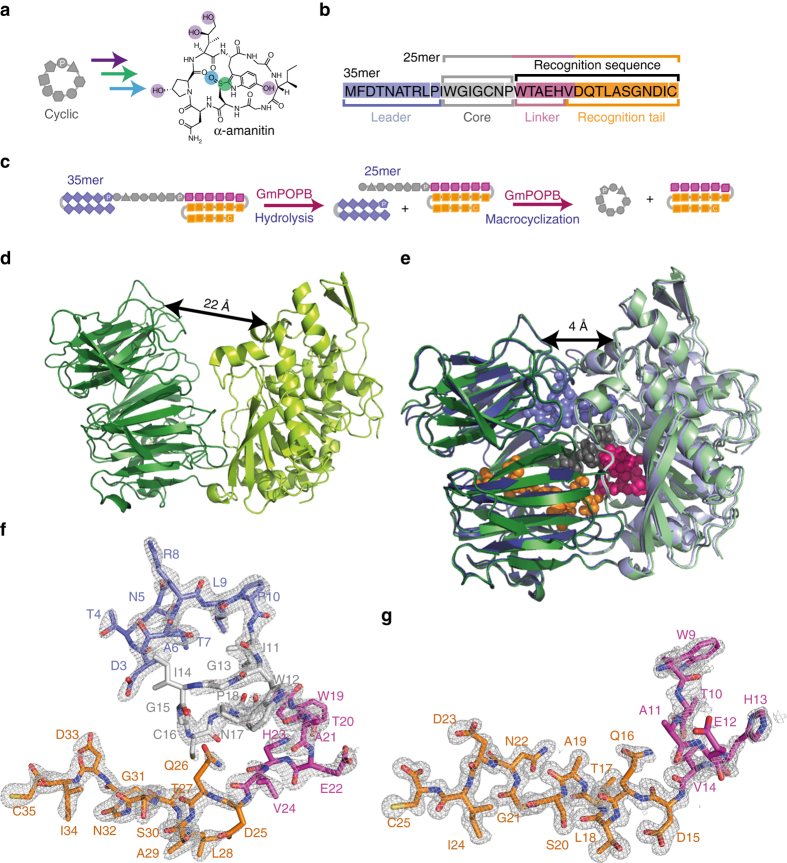
Amanitin biosynthesis and the role of GmPOPB. **a** Biosynthesis of α-amanitin from the cyclic peptide produced by GmPOPB involves oxidation (*blue circle*), hydroxylations (*purple circles*), and cross linking (*green circle*). Amino acids in the cyclic peptide are depicted as geometrical shapes, and the core proline is labeled. **b** 35mer substrate used by GmPOPB to catalyze peptide bond hydrolysis and macrocyclization. Peptide is divided in four regions, leader (*blue*), core (*light gray*), linker (*magenta*), and tail (*orange*). The color coding for peptide regions for **c**, **e**–**g** follows this. **c** Scheme of the reactions catalyzed by GmPOPB. **d** ApoGmPOPB in an open state showing 22 Å separation between the β-propeller (*dark green*) and the peptidase domain (*light green*). **e** Overlay of 25mer (*blue*) and 35mer (*green*) complexes of S577A. Separation between domains is reduced to 4 Å when peptide is bound. 35mer peptide is shown in *spheres*. *Darker colors* show the β-propeller domain, while *light colors* show the peptidase domain. **f** 35mer peptide bound to GmPOPB-S577A showing electron density (2*F*o—*F*c contoured at 1σ level in *gray mesh*) for peptide. **g** 25mer peptide bound to GmPOPB-S577A showing electron density for peptide (2*F*o—*F*c contoured at 1σ level in *gray mesh*). Supplementary Fig. 11 contains a stereo image depicting electron density maps for the 25mer and 35mer peptide complexes

In addition to PatG, there are four other broad classes of peptide macrocyclases^12, 14–16^ that operate through an acyl enzyme intermediate. The sortase class of enzymes, which catalyze transpeptidation by recognizing a C-terminal LXPTG motif^17^, the butelase enzyme, which is an asparagine/aspartate (Asx) peptide ligase^18^, the NRPS thioesterases^19^ and the prolyl oligopeptidase (POP) class of enzymes. A further important class of macrocyclases is that of the ATP-grasp superfamily, which as the name suggests rely on ATP hydrolysis to drive macrocycliation^20^. The enzymes that catalyze close to traceless peptide bond formation regardless of the peptide sequence—, i.e., only one residue from the precursor peptide recognition tag is carried over to the final cyclic product—are PatGmac family members, butelase, and POP macrocyclases. The POPB from Basidiomycete fungi such as *Amanita bisporigera* and *Galerina marginata* (GmPOPB) species have been reported as having *k*_cat_ values comparable to butelase, the fastest rate observed for peptide macrocyclisation^15, 21^. GmPOPB is the macrocyclase responsible for macrocyclization of amatoxins, eight amino-acid ribosomal peptides with the core sequence IWGIGC(N/D)P. Amatoxins are cyclic peptides further modified by a characteristic sulfoxide cross-link between tryptophan and a cysteine (Fig. 1a), and hydroxylation (the extent of which vary). The genomes of *G. marginata* and other amatoxin producing *Amanita* species possess more than 50 gene sequences annotated as AMA1 (amatoxin precursors) in which there is considerable diversity in the long C-terminal tail that follows the core sequence^15, 22^. Amatoxins are the cause for the toxicity of *Amanita* and *Galerina* mushrooms. They are readily absorbed through the gut, and a lethal dose in adults is <10 mg^23^. Amatoxins are stable to inactivation by either the mammalian digestive tract or cooking, thus consumption of even small numbers of such mushrooms is often fatal. Amatoxin toxicity arises from its accumulation in the liver where it inhibits RNA polymerase II leading to irreparable liver failure^23^. The highly stable and potent toxicity of amatoxins has led to their exploration as warheads for targeted cancer therapy^24, 25^. The amatoxin peptide precursor is produced as a 35 amino-acid linear substrate (Fig. 1b), which is first processed to a 25 residue peptide (25mer) by removal of the highly conserved 10 N-terminal amino-acid leader^26^ that is discarded. The newly exposed N-terminal eight residues of the 25mer product are then macrocyclized and the tail, which is necessary for macrocyclization, is discarded (Fig. 1c). Remarkably, both proteolysis and macrocyclization steps are carried out by the same enzyme, GmPOPB^15^.

We report the functional and structural characterization of GmPOPB and establish the molecular features that determine whether the enzyme catalyzes proteolysis or macrocyclization. Informed by structural and biophysical studies, we have designed a much simpler substrate with fewer C-terminal residues that can be macrocyclized by GmPOPB at synthetically useful rates. The shorter substrate is more cost effective to produce by solid-phase chemical synthesis allowing the generation of more chemically diverse macrocycles, a valuable biotechnological tool.

## Results

### Structural biology of Apo and substrate-bound GmPOPB

The apo protein crystals belong to space group *P*2_1_2_1_2_1_, with one monomer in the asymmetric unit. The structure was determined at 2.4 Å resolution by molecular replacement using the β-propeller domain of the proline oligopeptidase from porcine brain (residues 82–450, PDB:1h2z) as search model. The refined apo model (PDB:5N4F) includes residues 7–222, 230–695, and 704–726, and the missing regions are presumed to be disordered portions of the protein. The protein contains two domains as observed in other POP enzymes^27^. The domain containing the putative catalytic residues (Ser577, Asp661, His698) comprises residues 1–81 and 450–728, and the other domain is a seven bladed β-propeller, comprising residues 82–449 (Fig. 1d). In the apo structure, the two domains are in an “open” conformation, in an arrangement reminiscent of a hinged lid on a bottle. This open conformation has been observed in other POPs in crystal form when free of ligand^28, 29^. The catalytic serine sits at the tip of a loop and points toward the β-propeller domain (Supplementary Fig. 7). The side chain oxygen of Ser577 is 5.6 Å from the side chain carboxylate of Asp661; His698 is on a loop that is disordered. Ser577 and Asp661 of GmPOPB occupy the same position as Ser554 and Asp641 in the porcine proline oligopeptidase structure^30^.

In order to obtain co-complexes, we mutated each residue of the presumed catalytic triad (mutants S577A, D661A, and H698A) to ensure inactive protein. Crystals were obtained for the 35mer complex for S577A and H698A; with the 25mer substrate S577A and D661A (Table 1). GmPOPB-S577A (the higher resolution of the pair) bound to the full-length substrate (35mer) belongs to space group *P*2_1_ with four monomers in the asymmetric unit. For ease of discussion, we split the 35mer into four regions (Fig. 1b), the 10 residue leader (residues 1–10), the 8 residue core (11–18), 6 residue linker (19–24), and the 11 residue recognition tail (25–35). The refined model (PDB:5N4C) includes residues 6–225 and 228–727 of the protein and residues 3–35 of the peptide (Fig. 1c). The same inactive mutant of GmPOPB was used to obtain a complex structure with the 25mer substrate; it comprises core (residues 1–8), linker (9–14), and recognition tag (15–25). The refined model includes residues 4–727 of the protein and residues 9–25 (linker and recognition tag) of the peptide (PDB:5N4B). Although we observed residual difference electron density for the N-terminal residues of the 25mer, we were unable to satisfactorily model it. To observe interactions when the catalytic residue Ser577 is present, we also obtained complex structures of the H698A mutant bound to the 35mer peptide (PDB:5N4E) and the D661A mutant bound to the 25mer peptide (PDB:5N4D).

**Table 1:** Data collection and refinement statistics

	GmPOPB-S577A_25mer	GmPOPB-D661A_25mer	GmPOPB-S577A_35mer	GmPOPB-H698A_35mer	apo_GmPOPB
*Data collection*					
Space group	*P* 2_1_ 2_1_ 2_1_	*P* 2_1_ 2_1_ 2_1_	*P* 2_1_	*P* 2_1_ 2_1_ 2_1_	*P* 2_1_ 2_1_ 2
Cell dimensions					
*a*, *b*, *c*	99.1, 114.7, 141.3	99.0, 114.9, 141.3	100.8, 142. 6, 116.4	99.2, 115 141. 5	90.89, 105.62, 86.96
*α*, *β*, *γ*	90.0, 90.0, 90.0	90.0, 90.0, 90.0	90.0, 90.29, 90.0	90.0, 90.0, 90.0	90.0, 90.0, 90.0
Resolution	1.44 (1.49–1.44)	1.62 (1.678–1.62)	2.19 (2.268–2.19)	2.9 (3.0–2.9)	2.4 (2.49–2.4)
Unique reflections	288,485 (28,320)	204,059 (20,188)	163,136 (16,107)	32,664 (1690)	31,441 (2534)
Completeness (%)	99.7 (99)	100 (100)	97 (96)	97 (93)	99 (77)
Mean *I*/sigma (*I*)	9.3 (1.3)	6.9 (1.0)	6.7 (1.2)	6.8 (1.7)	14.5 (2.2)
*R*-merge	0.114 (1.01)	0.059 (0.69)	0.112 (1.0)	0.068 (0.53)	0.029 (0.56)
*R*-meas	0.063 (0.588)	0.084 (0.98)	0.27 (1.5)	0.096 (0.52)	0.036 (0.72)
Redundancy	6.0 (6.6)	6.7 (6.6)	2.0 (2.0)	1.8 (1.6)	5.1 (3.3)
*Refinement*					
Resolution range	43.3–1.44	46.9–1.62	82.3–2.19	32.0–2.9	45.44–2.4
Reflections used in refinement	288,428 (28,313)	203,960 (20,185)	163,135 (16,107)	32,664 (1690)	31,437 (2534)
*R*_work_/*R*_free_	0.17/0.19	0.20/0.23	0.23/0.26	0.255/0.303	0.21/0.25
Protein residues	1478	1478	3014	1484	703
RMS(bonds)	0.015	0.020	0.012	0.007	0.007
RMS(angles)	1.28	1.82	1.42	1.10	1.17
Ramachandran favored (%)	97	97	97	97	97
Ramachandran allowed (%)	2.5	2.5	3.1	2.9	2.3
Ramachandran outliers (%)	0.14	0.3	0.3	0.48	0.29
Rotamer outliers (%)	0.24	1.4	0.95	1.1	0.34
Clashscore	3.73	1.63	1.02	3.42	2.64
Average B-factor	15	21	15	32	44
Macromolecules	14	20	14	34	43
Solvent	27	31	43	37	38

**Fig. 2:**
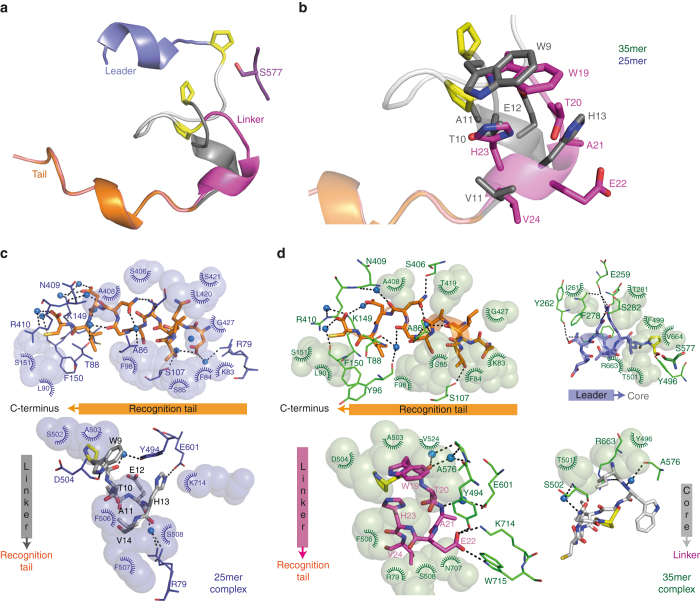
Interactions between GmPOPB and linker regions for 35mer and 25mer substrates are different. **a** Overview of bound peptides. Colors for distinct peptide regions are as in Fig. 1b, except that the linker in the 25mer complex is in *dark gray*. Position of the catalytic Ser577, and core and leader proline residues (*yellow*) are indicated in *sticks*. **b** Comparison of the linker region between 35mer (*magenta*) and 25mer (*gray*) complexes. Highlighted are the very different position of Glu and His in contrast to tryptophan, which in both structures occupies the same pocket although the conformation is different. **c** Interactions between peptide tail and linker of the 25mer peptide and GmPOPB-S577A. Residues within hydrogen bonding distance are shown in *lines*, residues participating in hydrophobic interactions are in *spheres*, water molecules are depicted as *small red spheres*, prolines are shown in *yellow*. Color coding for peptide substrate regions follows **a**, residues from GmPOPB are depicted in *blue*. **d** Interactions between peptide tail, linker, core, and leader (anti-clockwise) of the 35mer peptide and GmPOPB-S577A. The same representation as **c** is used except residues from GmPOPB are depicted in *green*

In all 35mer and 25mer complexes, the enzyme adopts the same “closed” conformation in which the lid (the propeller domain) sits on top of the catalytic domain (Fig. 1e). In both 25mer complexes the N-terminal residues of the substrate (IWGIGCN) are disordered; in the S577A–35mer complex the N-terminal two residues (MF) are missing, while in the H698A complex only the first N-terminal residue is absent. There are no large differences between the protein backbone positions in the complexes with the 35mer and 25mer substrates (root mean square deviation (rmsd) of 0.48 Å over 720 Cα positions for the S577A structures). There are also no major differences between the H698A–35mer and S577A–35mer complexes, and between the S577A–25mer and D661A–25mer complexes. Table 1 shows the data collection and refinement statistics for all structures. The recognition tail adopts an identical distorted 3_10_ helix conformation inserted into the middle of the β-propeller domain in both the 25mer and 35mer complexes (Fig. 1f, g and 2). The carboxyl terminus sits in a pocket where it makes water-mediated hydrogen bonds to the protein. To our surprise, there are only a few hydrogen bonds between the protein and the tail (Fig. 2c for the S577A–25mer complex and Fig. 2d for the S577A–35mer complex). Comparison to the apo structure reveals that binding of substrate induces no significant changes in the core of the propeller domain (rmsd of 0.75 Å over 360 Cα positions for the S577A structures). The changes that occur (relative to the apo structure) are in loops around the catalytic site.

Significant differences between the two substrates are observed for the residues of the linker region, since it occupies distinct conformations on the 25mer and 35mer complexes (Fig. 2b). V24 faces Arg79 and V14 is toward Phe506. H23 does not form any hydrogen bonds, while H13 is hydrogen bonding with Glu601. Tyr494 is within hydrogen bonding distance from E22, while on the 25mer complex Tyr494 is interacting with W9. This peptide twisting causes a tryptophan present in the peptides from both complexes (residue 19 in 35mer, 9 in the 25mer), C-terminal to the site of cleavage, to occupy a binding pocket close to the active site. The oxyanion-stabilizing Tyr496 is in close proximity to the core proline (P8) in the 25mer structure (D661A mutant), while Tyr496 hydrogen bonds with P18 in the 35mer complex (Fig. 2d).

Substrate residues I11–P18 (the core peptide is not seen in the 25mer, apart from weak density from P8 in the D661A–25mer complex) form a twisted loop, which makes contacts with the protein and within itself; atoms that will ultimately form the macrocycle are 7.6 Å apart (Fig. 2d). The core peptide interposes between substrate P18 and enzyme Ser577, which is over 8 Å away. Substrate P10, the site of proteolytic cleavage, is positioned for attack by Ser577 (the Cβ of the mutated residue is 3.3 Å from the carbonyl with plausible geometry). In the crystal structure of H698A–35mer complex, the hydroxyl of Ser577 is in hydrogen bonding distance from P10, in a position suited for nucleophilic attack but the structure is less ordered, notably the loop containing the mutation H698A. Tyr496 is on the opposite face of the carbonyl 2.8 Å from the oxygen and positioned to stabilize the tetrahedral intermediate from attack of Ser577 (Fig. 2d). Both the interaction and the role of Tyr496 are conserved in other POPs. In the 35mer complex structures, residues 2–9 adopt a helical arrangement that ends up exposed to solvent at the N-terminus and makes few contacts with the protein (Fig. 2d). In none of the GmPOPB structures that we have obtained are the catalytic residues arranged in the traditional manner, the closest approach of the His698 and Ser577 is 13 Å and residues block simple movement (Supplementary Fig. 7). To confirm the importance of the putative catalytic triad for GmPOPB, the mutants S577A, D661A, and H698A were evaluated for activity, and were inactive with both 25mer and 35mer substrates, using 5 μM GmPOPB and monitoring the reaction progress after 24 h.

### Mutations in the histidine loop decrease enzyme activity

To study other residues involved in hydrolysis and macrocyclization, additional mutants H698N, R663A, R663Q, R663K, and W695Δ (deletion) were generated. These mutants were designed based on comparison of sequence alignments between other POP enzymes and the very similar POPA enzyme from *G. marginata*, enzymes that solely act as proteases. Arg663 is highly conserved in POPs and thought to play a role in catalysis or substrate binding, since it makes hydrogen bonds with the peptide substrate^31^. Supplementary Fig. 9a depicts a sequence alignment comparing POPA, POPB, PCY1 (another macrocyclase from the prolyl oligopeptidase family), and porcine POP while Supplementary Fig. 9b depicts the position of these residues in GmPOPB. H698N was insoluble and not evaluated. The other mutants possessed diminished activities for both peptide bond hydrolysis and macrocyclization. The amount of cyclic peptide present after incubation for 16 h with the 25mer substrate was R663Q > R663A > W695Δ > R663K. When the 35mer was used as substrate, the mutants demonstrated diminished activity for peptide bond hydrolysis and almost undetectable activity for macrocyclization (Supplementary Fig. 9c).

### Kinetic characterization and substrate scope of GmPOPB

Previous analysis employed GmPOPB isolated from the *G. marginata* mushroom after transformation with *Agrobacterium tumefaciens* ^15^. We examined kinetic parameters and performed a substrate specificity study on the enzyme isolated employing a bacterial overexpression system. Substrates tested are shown in Supplementary Fig. 1. Our results on the native overexpressed enzyme confirm the previous findings^15^ obtained for protein purified from mushroom that the full-length 35mer substrate is cleaved and the resulting 25mer is released (Supplementary Fig. 3). The kinetic data for expressed protein with the 25mer but not 35mer have been previously reported^15, 21^. The 25mer then rebinds (in competition with the 35mer) for macrocyclization. Cleavage and macrocyclization do not occur in a single binding event^15^. The 25mer accumulates as an intermediate although the proteolysis reaction is slower than cyclic peptide formation (Supplementary Fig. 2). Very similar values for *K*_m_ and *k*_cat_ were obtained for all full-length substrates evaluated, with *K*_m_ values ranging from 8 to 51 μM, while *k*_cat_ was between 3.2 and 35 min^−1^ (Supplementary Fig. 2; Supplementary Table 2). Conservative amino-acid substitutions within the peptide substrate had no effect either on kinetic parameters or yield of cyclic product. Less conservative substitutions such as mutation to alanine or 9mer core (IWGIGC**A**NP the bold underlined residue represents the insertion) led to reduced macrocyclization and increased linear peptide, the product of peptide hydrolysis instead of macrocyclization (Supplementary Fig. 8).

### Equilibrium binding of substrates and products

**Fig. 3:**
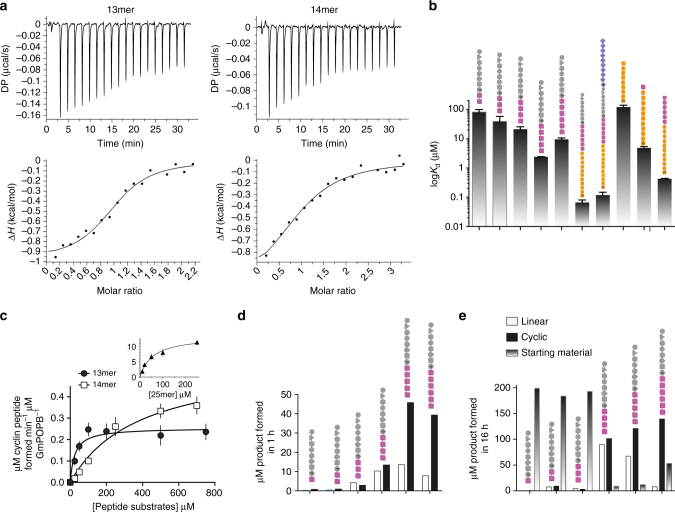
Binding and kinetics of substrates and recognition tail truncations. **a** ITC binding curves for the 25mer peptide (obtained with the S577A mutant) and the 17 amino-acid recognition (linker and tail) sequence. **b** *K*_d_ values of peptides examined by ITC shows the 35mer and 25mer bind with similar affinity, the leader does not bind, the tail on its own binds weakly and the contributes to binding energy. The peptides are colored as Fig. 1c with N-terminus at top. *Error bars* are standard error of the mean from the average of at least two independent measurements (Supplementary Table 3). **c** Michaelis–Menten curves show both the 13mer and 14mer are substrates for the enzyme. Fitted values for *K*_d_, *K*_m_, and *k*_cat_ are shown in Supplementary Tables 2 and 3. *Error bars* are standard error of the mean from duplicate measurements. **d** Cyclic peptide produced after 1 h reaction with 1 μM GmPOPB and 200 μM of various peptide substrates. **e** Peptide produced after 16 h of reaction with 1 μM GmPOPB and 200 μM of various peptide substrates. Peptide sequences are colored as Fig. 1b and detailed in Supplementary Fig. 1

Binding of the inactive mutant S577A to the 25mer, 35mer, a series of truncated substrates (10mer–14mer), as well as the recognition sequence (17mer) WTAEHVDQTLASGNDIC, the truncated recognition sequences VDQTLASGNDIC and TLASGNDIC, and the leader peptide MFDTNATRLP were evaluated by isothermal titration calorimetry (ITC). The results of S577A with both the 25mer and recognition sequence have previously been reported^21^. Binding of H698A to the 25mer was also measured. The only peptide showing no detectable binding at concentrations up to 1 mM was the 10-residue leader peptide MFDTNATRLP. The full-length substrates and products displayed tight binding (*K*_d-25mer–S577A_ = 67 ± 14 nM^21^, *K*_d-25mer–H698A_ = 47 ± 11 nM, *K*_d-35mer_ = 120 ± 30 nM, *K*_d-recognition_ = 430 ± 10 nM^21^; binding is dominated by enthalpic contributions (Supplementary Fig. 6b) (Fig. 3a shows representative ITC traces for the 13mer and 14mer substrates, Fig. 3b shows *K*_d_ values for all peptides evaluated, Supplementary Fig. 10 shows raw data for all binding curves). The inactive mutant H698A has identical *K*_d-25mer_ to the S577A mutant suggesting the lack of activity results from catalytic incompetence rather than disruption of substrate binding. Interestingly, despite being longer and having the potential for more interactions with the protein, the 35mer peptide shows slightly weaker binding compared to the 25mer, mostly due to decreased Δ*H*. A comparison of the complex structures shows that in the 35mer complex there is disorder of side chains in the segment TAEHVD (linker region) but not in the 25mer. To investigate the role of recognition tag peptides corresponding to the entire recognition sequence (linker plus tail, WTAEHVDQTLASGNDIC—17 residues), the recognition tail plus the valine from the linker (VDQTLASGNDIC 12 residues) and the highly conserved portion of the tail (TLASGNDIC 9 residues) were tested for binding and gave *K*_d-17mer–recogSeq_ = 0.43 ± 0.01 μM^21^, *K*_d-12mer–recogSeq_ = 5 ± 1 μM and *K*_d-9mer–recogSeq_ = 121 ± 19 μM (Supplementary Table 3; Supplementary Figs. 6 and 10). Previously, we showed that the recognition sequence dominates binding, with a difference in Δ*G* of only 1.34 kcal mol^−1^ between the 17mer recognition sequence and the 25mer peptide^21^. To explore how much contribution to the binding energy comes from the linker region, we evaluated the binding of truncated recognition sequences. Our data show that the linker region is important in binding as the loss of the linker (shrinking the recognition sequence from 17 to 12 amino acids) reduces binding affinity 20-fold. On its own, the highly conserved nine-residue tail bound rather weakly, consistent with the few interactions observed with the protein. Following from this finding, a series of truncated peptides (core plus parts of the linker) were tested and revealed a trend in which binding affinity increased from 10mer to 13mer (*K*_d-10mer_ = 83 ± 17 μM, *K*_d-11mer_ = 39 ± 18 μM, *K*_d-12mer_ = 21 ± 5 μM, *K*_d-13mer_ = 2.4 ± 0.1 μM) peptides, but decreased slightly with the 14mer peptide (*K*_d-14mer_ = 9.5 ± 1.1 μM) (Fig. 3b); the 9mer was not sufficiently soluble for analysis. We noted that the difference in affinity between the 35mer and the core plus linker (13mer) was ~20-fold.

## Discussion

GmPOPB can form and hydrolyze peptide bonds depending on the substrate length and structure. Both reactions proceed by similar chemical mechanisms, passing through an acyl enzyme intermediate. Typical POPs catalyze peptide bond cleavage following a proline, and less efficiently an alanine, showing strong preference for substrates shorter than 30 amino acids^32^. GmPOPB is unusual in that it processes a 35 amino-acid substrate, the longest observed for a POP. POP enzymes possess an aspartate, histidine, and serine catalytic triad. Molecular dynamic simulation studies comparing the porcine and bacterial POPs have proposed a mechanism in which inter-domain “breathing” is required for catalysis^33^. Although structures of apo and POPs bound to short peptide substrates and inhibitors (ranging from 2^30^ to 7 amino acids^34^) are available, no substrate complex with a long peptide has previously been determined. Interestingly, in GmPOPB complexes the residues of the presumed catalytic triad are not aligned. His698 is 12 Å away (much further than in any other POP, Supplementary Fig. 7) from Ser577 but is essential for catalysis rather than binding. The enzyme–substrate complex shows that, apart from His698, there is no other residue in proximity to the active site capable of acting as general acid/general base with p*K*_a_ values near to the 8.0 measured from kinetic analysis^21^. The domain breathing motion similar to, but larger than, other POPs could correctly position the His698. Mutations of the highly conserved Arg663 and deletion of Trp695 (an insertion relative to other POPs, Supplementary Fig. 9a), which we predicted would affect loop structure and dynamics, were severely compromised in activity with both 25mer and 35mer substrates, consistent with our prediction. We cannot, however, exclude the possibility that His698 may be required for positioning toward a productive conformation of the enzyme–substrate complex rather than act as a base per se. The histidine loop is disordered on the H698A mutant structure and the H698N mutant is insoluble, hinting at a stabilizing role for His698. Analogous to lipid acyl hydrolases^35^, the enzyme would function with a catalytic dyad in which Ser577 is activated by a water molecule bridged to Asp661.

Comparison of the three-dimensional structures of the enzyme in complex with 25mer and 35mer substrates reveals only minor rearrangements of the protein, mostly in loops that accommodate the longer substrate. Both 35mer (proteolysis) and 25mer (macrocyclization) substrates bind to GmPOPB with high affinity driven mainly by enthalpy (Fig. 3b, Supplementary Fig. 7; Supplementary Table 3). Consistent with the observation of similar binding affinities of the 25mer and 35mer (*K*_d_ 67 and 120 nM, respectively)^21^, the 10-residue leader (only present in 35mer) does not bind. In both the 25mer and 35mer complex structures, the recognition tail (C-terminal 11 residues) is embedded deeply into the β-propeller domain in an essentially identical arrangement. The linker region, however, adopts very different arrangements in the two complexes, thus its interactions with the protein are quite distinct in the two structures (Fig. 2). ITC measurements show that the linker region, particularly the portion following the core peptide, makes substantial contribution to the binding energy. This is in contrast to the heterocyclase class of RIPP enzymes, where the linker plays no role and can be varied^36^. Our data show that the structure of the linker is important in binding and determines the orientation of the substrate at the active site (thus its fate).

**Fig. 4:**
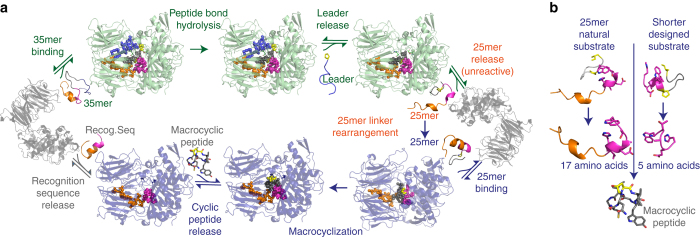
GmPOPB catalyzes peptide bond hydrolysis and macrocyclization. **a** The sequence of events catalyzed by GmPOPB starting with the 35mer peptide substrate. Peptide bond hydrolysis yields the leader sequence (which dissociates) and the 25mer peptide. The enzyme-bound 25mer is conformationally trapped and macrocyclization cannot proceed. The 25mer must dissociate to rearrange and re-bind only then does macrocyclization occur. Apo enzyme is shown in *gray*, the enzyme functioning as a protease is colored *green*, and functioning as a macrocyclase in *blue*. The peptide is colored as Fig. 1b. **b** A 25mer substrate yields a 17 amino-acid by-product, whereas the 13mer substrate generates a 5 amino-acid by-product, a much more economically efficient reaction. Substrates are colored as Fig. 1b

Previous kinetic assays and ours reveal that after removal of the leader, the remaining 25mer is released from GmPOPB, then it rebinds and undergoes the macrocyclization reaction^15^. In the 35mer complex, the core and linker adopt a tightly packed arrangement that is wedged between the active site loops. We conclude that the linker and/or core are unable to refold to the conformational arrangement seen in the 25mer complex (required for macrocyclization) in situ on a timescale comparable to dissociation. We propose the arrangement of and interactions between C-terminal 25 residues and protein that are seen in the 35mer complex act as a kinetic trap, which can only be escaped by dissociation (Fig. 4a). Conformationally trapped peptide reaction intermediates have been identified in other systems. For example, inhibition of proteases by serpins is accomplished by a suicide substrate mechanism, in which the complex is trapped in an inactive arrangement^37^.

Having identified the key role of the linker, we predicted that it should be possible to design simpler macrocyclization substrates that lacked the recognition tail. This would be valuable since the use of 25mer substrates to make eight residue macrocycles is not economic. ITC shows a 10-fold reduction in binding from 35mer to 13mer, kinetic analysis reveals the 13mer substrate has a *K*_m_ (25 μM) within error to the 25mer substrate (50 μM), while the 14mer possesses higher *K*_m_ (380 μM). Similar *k*_cat_ values were observed with both shorter substrates (0.49 and 0.58 min^−1^ for the 13mer and 14mer, respectively, Fig. 3c; Supplementary Table 2) but these are smaller than the 25mer (18 min^−1^). Linear peptide (product of hydrolysis instead of macrocyclization) was observed when shorter peptides were utilized as substrates (Fig. 3d) consistent with the linker playing a key role in substrate positioning. After 16 h of reaction both 13mer and 14mer substrates produce similar amounts of macrocycle, but the 14mer generates less linear product. Linear peptide produced this may not be a significant drawback, as purification of macrocycles from liner peptides is straightforward^12, 38^. Compared to PatG this represents a significant improvement, since biocatalytic reactions with PatG in vitro can require over 7 days and utilize up to stoichiometric amounts enzyme^12, 38^.

GmPOPB is an unusual enzyme catalyzing, depending on substrate length, proteolysis, or macrocyclization using the same catalytic machinery (Fig. 4a). Further complexity comes from the fact that GmPOPB itself generates its 25 residue macrocyclization substrate. The internal structure of the substrate is critical to how the enzyme binds the substrate and to which reaction is catalyzed. As a consequence of this requirement for a specific substrate structure, the enzyme must release the 25mer peptide, allowing it to refold rather than simply moving to the second reaction in a processive manner. Previous work had identified the crucial nature of the recognition sequence in the substrate, but suggested its full length was a requirement for macrocyclization. Our structural work supported by calorimetry and kinetics reveals that shorter peptides are suitable substrates, if their design preserves important interactions with the protein and maintains the peptide structural recognition. GmPOPB recognizes residues within the linker connecting the core and the recognition tail, and this recognition is critical to position the substrate for macrocyclization. A substrate with five or six C-terminal residues (as opposed to 17) chosen to mimic the linker can be efficiently macrocyclized at synthetically useful rates (Fig. 4b). This work highlights the power of structural and mechanistic studies to redesign substrates or enzymes for use in biotechnology.

*Note added in proof:* Since the submission of this manuscript two papers were published studying POPs, further demonstrating the importance of this class of macrocyclase enzymes. One reports the structure of the related PCY1 enzyme^55^ and the other discusses broadening of the substrate profile of GmPOPB^56^.

## Methods

### Materials

Peptides were purchased from Biosynthesis, as free amine and free carboxylic acids, at a purity >90%. Buffers and chemicals, unless specified, were from Sigma.

### Expression of recombinant proteins

The plasmid pJExpress414 encoding the codon optimized *G. marginata POPB* gene was purchased from DNA 2.0. Plasmids were transformed into BL21(DE3) cells (Agilent). Cultures (50 mL) were grown overnight at 37 °C in the presence of 100 μg/mL ampicillin, then diluted 100-fold into 6 L Terrific Broth (TB) media. These cultures were grown at 37 °C with shaking (200 rpm) until the optical density at 600 nm (OD600) reached 0.6. Cells were cooled down for 1 h to 16 °C, and protein expression was then induced by the addition of 0.5 mM isopropyl β-d-thiogalactopyranoside (IPTG, Generon). Cultures were incubated for an additional 16 h and centrifuged at 6000×*g* for 15 min. Cell pellets were resuspended in 250 mL Ni–NTA lysis/wash buffer A (50 mM HEPES pH 8.0, 300 mM NaCl, 10 mM imidazole, 10% glycerol, and 2 mM β-mercaptoethanol) supplemented with complete EDTA-free protease inhibitor tablets (Roche Applied Science). The resulting suspension was lysed by two passages through a cell homogenizer at 30,000 psi, and purified by nickel chromatography. Each desired protein was eluted using a step elution with lysis buffer supplemented with 250 mM imidazole (buffer B). Eluted protein was dialyzed overnight against buffer C (50 mM HEPES (pH 8.0), 50 mM NaCl, 10% glycerol, and 2 mM β-mercaptoethanol) while simultaneously the His-tag was cleaved by TEV protease (prepared in house^39^). This dialyzed TEV-cleaved mixture was loaded onto a Histrap column connected in tandem to a Hitrap Q-FF column. Both columns were washed with buffer C, and GmPOPB was eluted during this wash. Fractions were pooled and concentrated to <8 mL (at 10 mg/mL approximately). Protein was loaded onto a Superdex S200 gel filtration column (GE Healthcare) pre-equilibrated with storage buffer D (50 mM HEPES (pH 8.0), 50 mM NaCl, 10% glycerol, and 2 mM β-mercaptoethanol). Fractions containing pure protein were combined, concentrated, divided in aliquots, flash frozen, and stored at −80 °C. Protein concentrations were determined by absorbance at 280 nm^40^.

### Site-directed mutagenesis

Mutants S577A, D661A, H698N, R663A, R663Q, R663K, and W695Δ were generated by a published mutagenesis protocol^41^. Oligonucleotides for mutagenesis were purchased from IDT. Sequences of primers used for mutagenesis and sequencing are given in Supplementary Table 1. Sequencing was performed using at least three primers to cover the entire gene sequence (Eurofins).

### General procedure for kinetic assays

Comparison between distinct substrates was performed in 50 mM Tris pH 8.0, 50 mM NaCl, 10 mM DTT with varying concentrations of substrates at room temperature. All reactions were performed in duplicates. Reactions were started by adding GmPOPB (50 nM for GmAMA1_C6S, 1 μM for the 13mer and 14mer, and 20 nM for other substrates) to the assay mixture containing buffer and peptide. Reactions were quenched at several time points by adding 50 μL reaction mixture to 20 μL 6% TFA. Reactants were separated from products for quantification by injecting 50 μL of each quenched time point mix onto a ZORBAX SB-C18, 5 µm, 9.4 × 50 mm (Agilent) column connected to an Agilent LC-MS (G6130B Single Quad, Agilent Technologies). Reactants were separated from products using a gradient from H_2_O containing 0.1% TFA or 0.1% formic acid and 5% acetonitrile to 50% acetonitrile, at 1.5 ml/min for 8 min. Peaks with ultra violet (UV) absorbance at 220 and 280 nm were integrated, the area of peaks corresponding to reactant and products was used to calculate the percentage of product formed after a correction for differences in the extinction coefficient of each peptide was applied (*ε*_280–25mer_ = 11,000 M^−1^ cm^−1^, *ε*_220–25mer_ = 46,000 M^−1^ cm^−1^, *ε*_280–cyclic_ = 5500 M^−1^ cm^−1^, *ε*_220–cyclic_ = 34,000 M^−1^ cm^−1^, and *ε*_280–tail_ = 5500 M^−1^ cm^−1^). The sum of product+substrate was assumed equal to the total initial amount of substrate, product converted from % to concentration. This value was divided by concentration of enzyme present to yield *v*/*E*_t_ (min^−1^). When enzyme mutants and peptides containing alanine in the core sequence were tested for activity, higher concentrations 5 μM enzyme and 100 μM substrate were incubated for 1 and 18 h at room temperature. For progress curves with the 35mer substrate, measurements were triplicate and quantification relied on ion counts from mass spectrometry. Mass signals corresponding to 35mer (1282.9 Da—M+3H), 25mer (900.7 Da—M+2H), leader peptide (1165.5 Da—M+H), recognition sequence (930.4 Da—M+2H), cyclic peptide (841.3 Da—M+H), linear peptide (859.4 Da—M+H) were monitored, the area of each was integrated and quantified using a calibration curve performed with the 25mer, 35mer, cyclic, and linear peptides as standards. Authentic cyclic peptide was quantified by UV absorbance. Data showing products formed after 1 and 16 h with truncated peptides were performed twice. UV and ion count approaches gave similar results for the 25mer. Kinetic data were fitted to a Michaelis–Menten equation using GraphPad Prism, and values reported are average and standard error of the mean.

### Isothermal titration calorimetry

All titrations were performed on a MicroCal PEAQ-ITC instrument (MicroCal, Malvern Instruments, Northamption, MA, USA) and the results were fitted with PEAQ-ITC analysis software (MicroCal, Malvern Instruments, Northampton, MA, USA). Peptide ligand solutions were prepared in 20 mM Tris pH 8.0 containing 1 mM DTT, prior to buffer exchange by three cycles of dilution in 50 mM Tris pH 8.0 with 50 mM NaCl, 10 mM DTT followed by concentration using a Microsep Advance centrifugal device equipped with a 1 kDa cut off membrane (Pall Corporation). The same three cycles of dilution in 50 mM Tris pH 8.0 with 50 mM NaCl and 10 mM DTT followed by concentration were performed with the protein to be used in the titration using a Vivaspin protein concentrator spin column with a 30 kDa cut off (GE Healthcare). A final dilution to the concentration to be used for titration was performed using the buffer that passed through during the protein buffer exchange, both for the protein and peptide to be used to avoid any possible buffer mismatch. The stirred cell contained 300 μL of protein (the inactive mutant GmPOPB_S577A at 20 μM for 35mer, 36 μM for 10mer, 36 μM for 11mer, 29 μM for 12mer, 42 μM for 13mer, 29 μM for 14mer, 37 μM for 9mer recognition sequence, 21 μM for 12mer recognition sequence), and the injection syringe contained 75 μL of peptide ligand (200 μM for 35mer, 924 μM for 10mer, 761 μM for 11mer, 484 μM for 12mer, 442 μM for 13mer, 582 μM for 14mer, 1 mM for 9mer recognition sequence, 677 μM for 12mer recognition sequence). Titrations of peptide into protein solutions were conducted at 20 °C. For all the titration experiments, a total of 19 injections of 2 μL were made at 120 s intervals. The heat released due to the first injection (0.4 μL) was excluded from data analyses. Binding data with the H698A mutant were performed by titrating enzyme (319 μM stock) into 25mer peptide (27 μM). Blank runs in which peptide (or H698A) was titrated into buffer were performed to correct for the heats of dilution and mixing, and the dilution isotherm for each peptide ligand was subtracted from the respective binding isotherm prior to curve fitting. Equilibrium dissociation constants (*K*_d_) as well as Δ*H* and Δ*S* values for binding of each peptide to protein were obtained by fitting the calorimetric data with a single-site model using the stoichiometry parameter *n* fixed at 1.0 using Malvern PEAQ-ITC data analysis software. The ITC data for S577A with both the 25mer and recognition sequence (17mer) have previously been published^21^. We performed all ITC binding experiments at least in duplicate, and calculations of average and standard error of the mean were performed with GraphPad Prism.

### Structural biology

ApoGmPOPB crystals were obtained by vapor diffusion at 20 °C using the hanging drop method. The initial conditions in the drop were 100 mg/mL GmPOPB, 30% PEG4000, and 100 mM MES buffer, pH 6.5. Several crystal clusters appeared after incubation at 20 °C for 1 week, which were crushed and used for microseeding using a 80 mg/mL GmPOPB solution and the same precipitant. Crystals were cryoprotected by addition of 10% glycerol to precipitant solution, and flash cooled in liquid nitrogen. All complex structures were obtained by vapor diffusion at 20 °C using the sitting drop method. Complexes with both 25mer and 35mer peptides were obtained by co-crystallization of 100 mg/ml protein and two-fold molar excess of peptide, and contained 12.5 mM Hexammine cobalt chloride as additive. For the S577A–25mer complex, crystals were obtained with 28% PEG6000, 100 mM Bicine pH 9.0, 60 mM magnesium formate, and 2.42% DMSO. Crystals were cryoprotected by addition of 12% glycerol to precipitant solution, and flash cooled in liquid nitrogen. For the D661A–25mer complex, crystals were obtained with 28% PEG6000, 100 mM Tris pH 8.3, and 90 mM sodium/potassium phosphate. Crystals were cryoprotected by the addition of 12% glycerol to precipitant solution, and flash cooled in liquid nitrogen. Crystals of S577A–35mer complex were obtained with 28% PEG6000, 100 mM Bicine pH 8.7, 64 mM sodium potassium phosphate. Crystals were cryoprotected by addition of 12% glycerol to precipitant solution, and flash cooled in liquid nitrogen. For the H698A–25mer complex, crystals were obtained with 27% PEGMME2000, 90 mM Bicine pH 8.7, and 100 mM potassium thiocyanate. Crystals were cryoprotected by addition of 13% glycerol to precipitant solution, and flash cooled in liquid nitrogen.

Data were collected at 100 K at the European Synchrotron Radiation Facility (ESRF) beamline ID30A-3 (S577A–25mer complex), Diamond Light Source beamlines I02 (apo GmPOPB), I04-1 (S577A–35mer complex), I03 (D661A–25mer), or in house on a Rigaku 007HFM rotating anode X-ray generator with a Saturn 944 CCD detector (H698A–35mer). Data were processed with HKL2000^42^ (S577A–25mer and H698A–35mer complexes) or Xia2-DIALS^43^ (apo GmPOPB, S577A–35mer and D661A–25mer complexes). All structures were solved by molecular replacement with PHASER^44^, followed by density improvement using PARROT^45^, then automatic building using Buccaneer^46^ and Arp/wARP^47^. Manual rebuilding was performed with COOT^48^, and refinement was performed with REFMAC5^49^ implemented in the CCP4 program suite^50^, Phenix^51^, and PDB_REDO^52^. Structural figures were generated with PyMOL (DeLano Scientific, LLC). In Fig. 4 the solution structures for 13mer, 25mer, and 35mer free were generated by PEPFOLD^53, 54^ and the macrocyclic peptide was adapted from α-amanitin (PDB: 3CQZ).

### Data availability

Sequences and plasmids for all clones used in this study have been deposited on Addgene, with the following IDs: 92234 (GmPOPB-Wild type), 92235 (GmPOPB-S577A). 92236 (GmPOPB D661A), 92237 (GmPOPB-H698A), 92238 (GmPOPB-R663A), 92239 (GmPOPB-R663K), 92240 (GmPOPB-R663Q), 92241 (GmPOPB-W695Δ), and 92242 (GmPOPB-H698N). Coordinates have been deposited in the Protein Data Bank, with accession codes 5N4B (S577A mutant bound to 25mer peptide), 5N4C (S577A mutant bound to 35mer peptide), 5N4D (D661A mutant bound to 25mer peptide), 5N4E (H698A mutant bound to 35mer peptide), and 5N4F (apoGmPOPB). All the other data supporting the findings of this study are provided within the article and supplementary files, and available from the corresponding author upon reasonable request.

## Electronic supplementary material

Supplementary information
