# Supplementary material for: Characterization of a dual function macrocyclase enables design and use of efficient macrocyclization substrates
Source: Nat Commun. 2017 Oct 19;8:1045. doi: 10.1038/s41467-017-00862-4 (PMC5648786; doi:10.1038/s41467-017-00862-4)
Supplement: Supplementary file 1 — Supplementary information [file 41467_2017_862_MOESM1_ESM.pdf]

Description of Supplementary Files

File Name: Supplementary Information

Description: Supplementary figures, supplementary tables, supplementary references

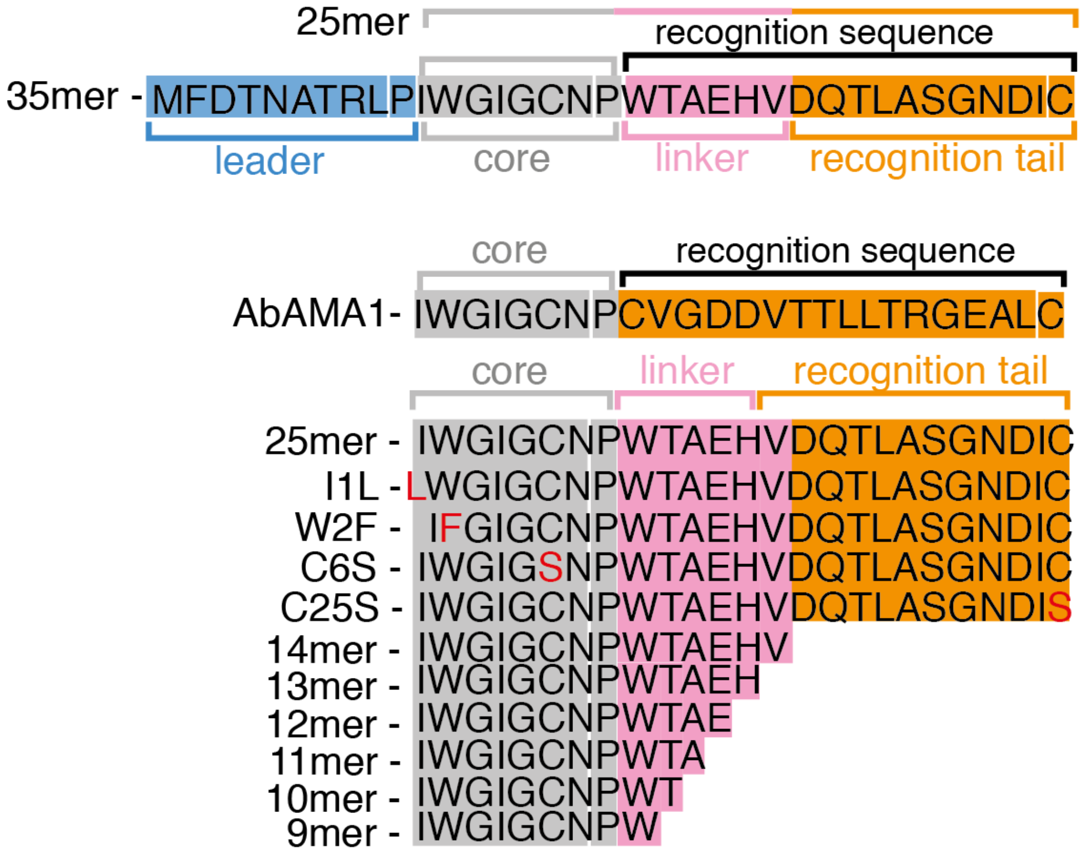

Supplementary Figure 1: Substrates tested with GmPOPB

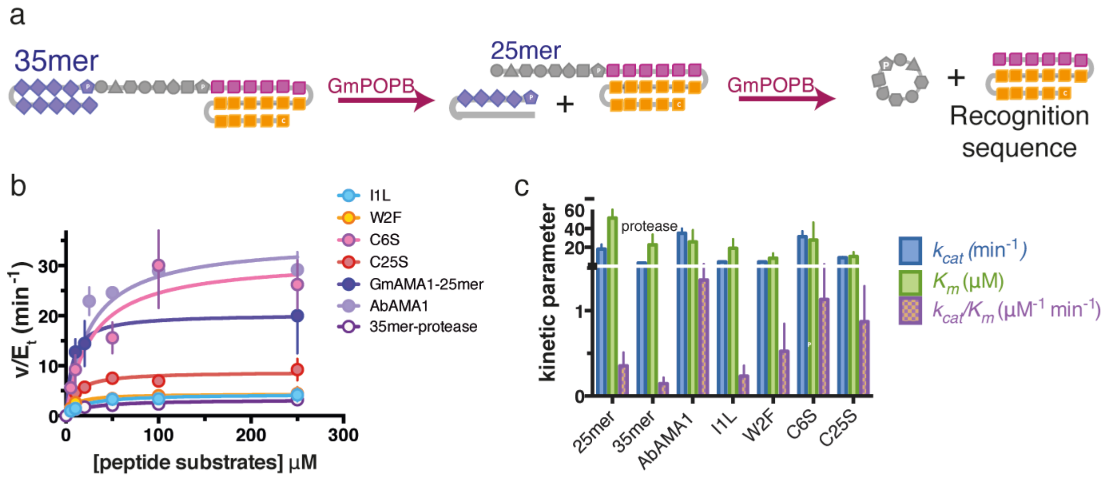

Supplementary Figure 2 25mer and 35mer substrates tested with GmPOPB.
(a) Reaction scheme.
(b) Michaelis-Menten curves for various substrates tested in the reaction. Nomenclature is as described in Supplementary Figure 1.
(c) Kinetic parameters obtained after fitting data to a hyperbolic Michaelis-Menten equation.
For b and c, error bars are standard error of the mean from duplicate measurements.

When the 35mer substrate was used, peptide bond hydrolysis was monitored (35mer-protease), while for 25mer substrate kinetic parameters for macrocyclization were determined. The data for the expressed protein with the 25mer substrate have been published previously.

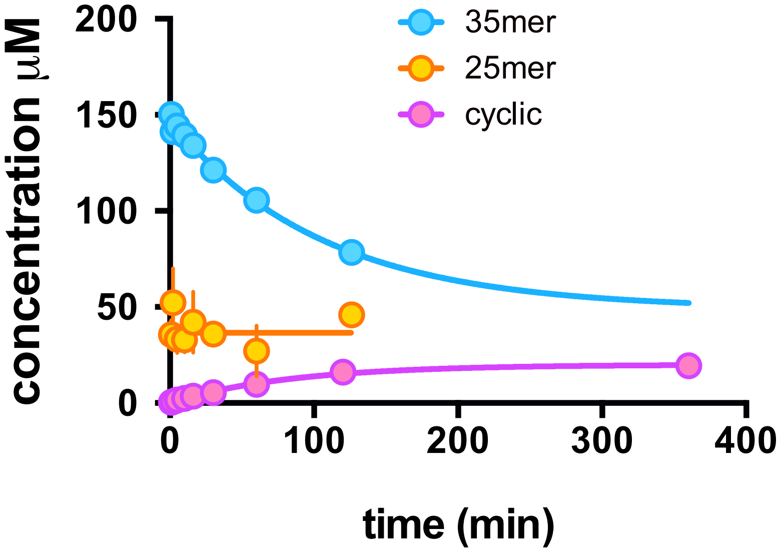

Supplementary Figure 3 Progress curve with the 35mer substrate showing μM concentrations of 25mer peptide, as well as 35mer consumption resulting in cyclic peptide formation. Lines are fits to a single exponential equation. Error bars are standard error of the mean from duplicate measurements.

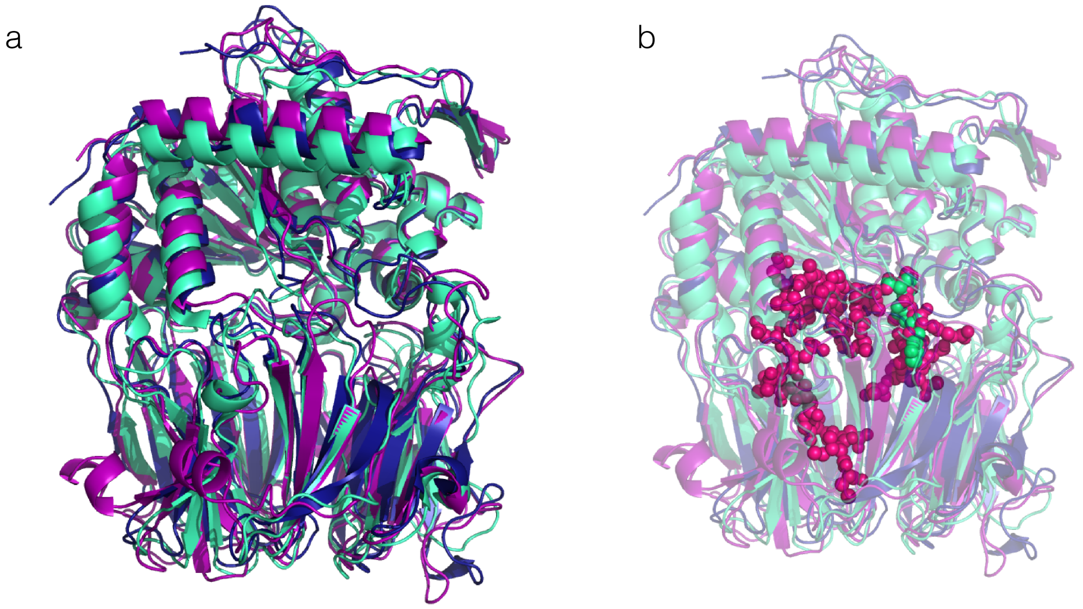

Supplementary Figure 4 Comparison of POP proteases and GmPOPB showing remarkable similarity despite small sequence conservation and distinct functions. Structures overlaid are from porcine POP (37% sequence identity, pdb: 1o6g – blue), *Myxococcus xanthus* POPB (31% sequence identity, pdb: 2bkl – cyan), and GmPOPB (purple). a) Overlay of structures omitting ligand. b) Overlay of structures showing bound peptides in cyan (2bkl) and pink (GmPOPB), showing they occupy a similar binding region but the peptide ligand is significantly longer in GmPOPB.

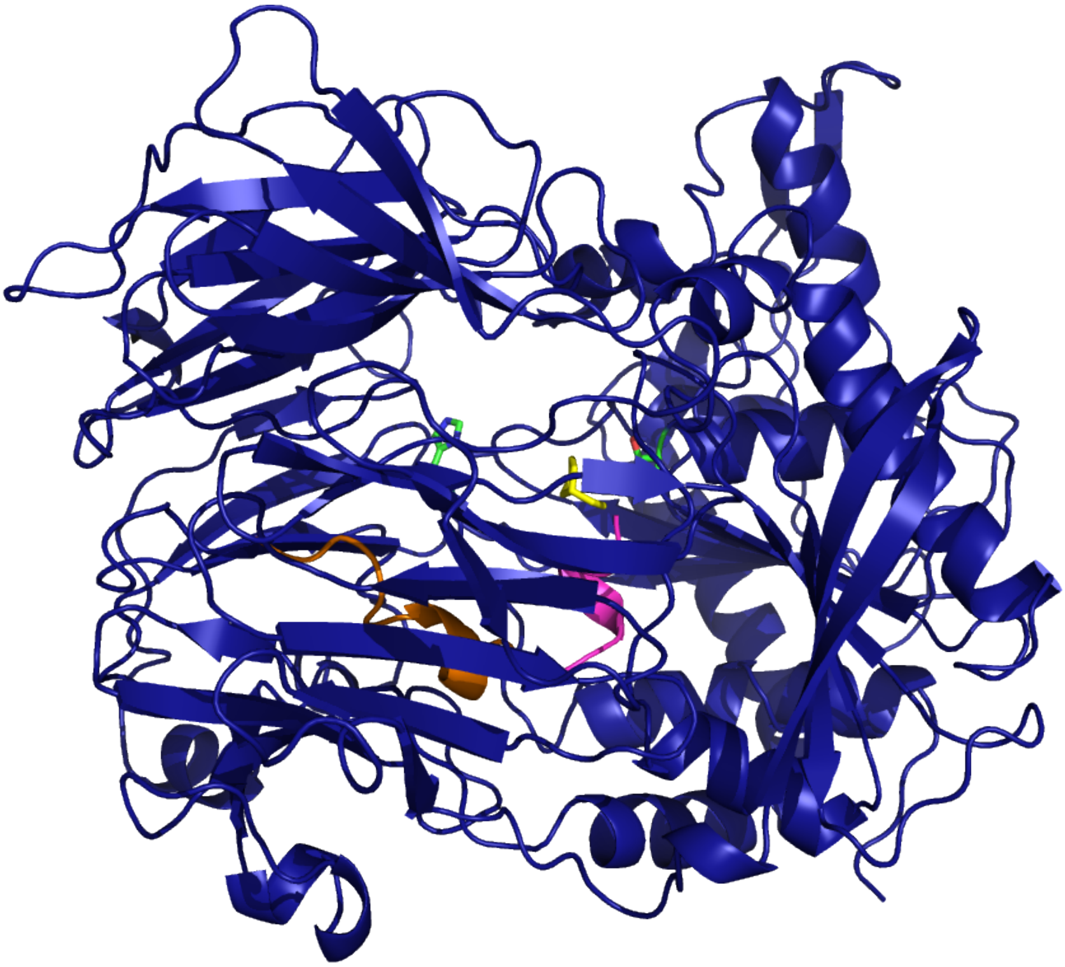

Supplementary Figure 5: crystal structure of D661A mutant bound to 25mer peptide showing overall structure. Peptide is colored as in Figure 1, catalytic triad is in green, core peptide proline is in yellow.

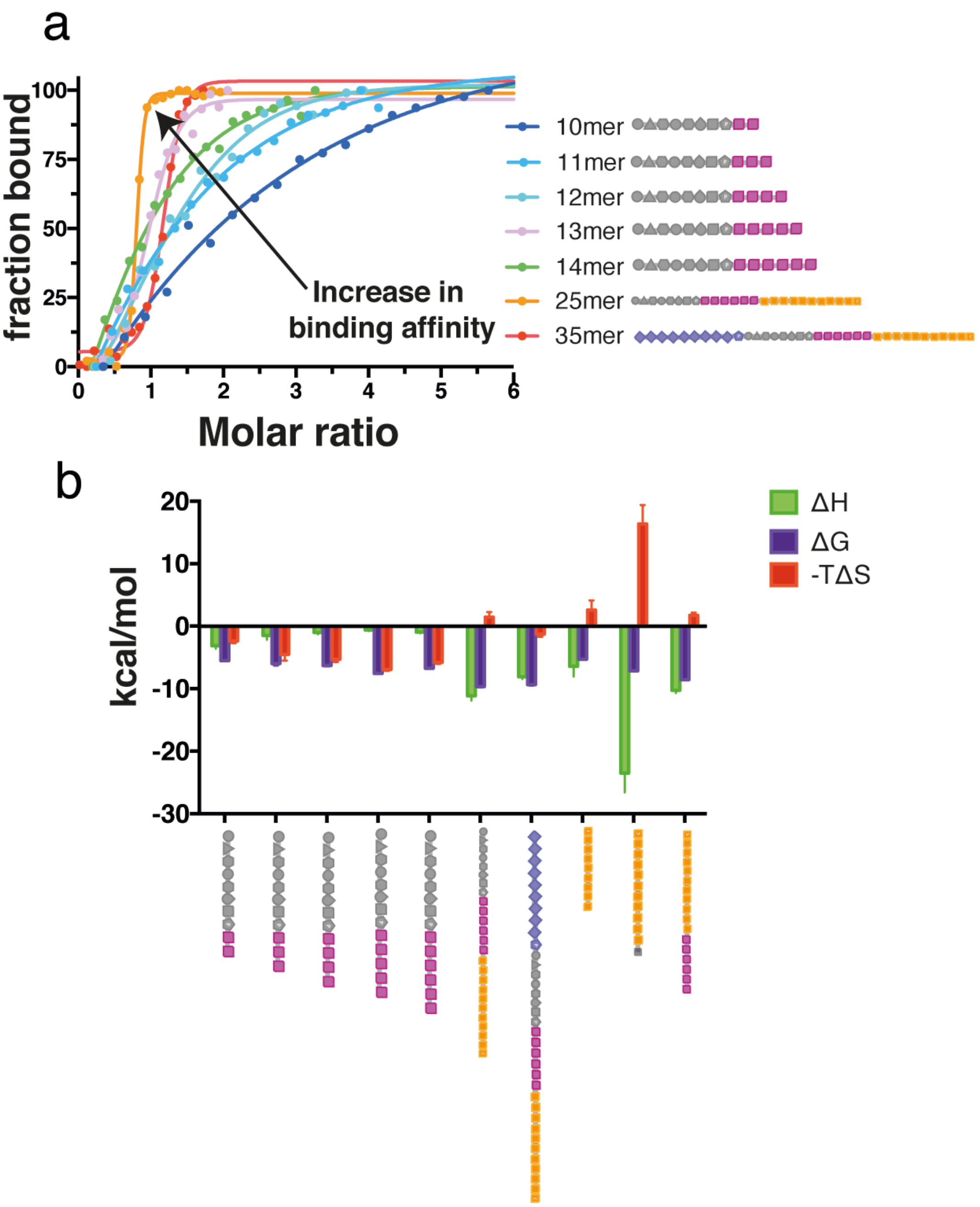

Supplementary Figure 6: ITC binding data for different peptides to the inactive S577A mutant of GmPOPB. Experimental conditions are described in Materials and Methods. a) Comparison of binding affinities of different substrates. b) Thermodynamic parameters for all peptides tested. Raw ITC traces for all peptides are shown in Figure S10. Error bars are standard error of the mean from the average of at least two independent measurements.

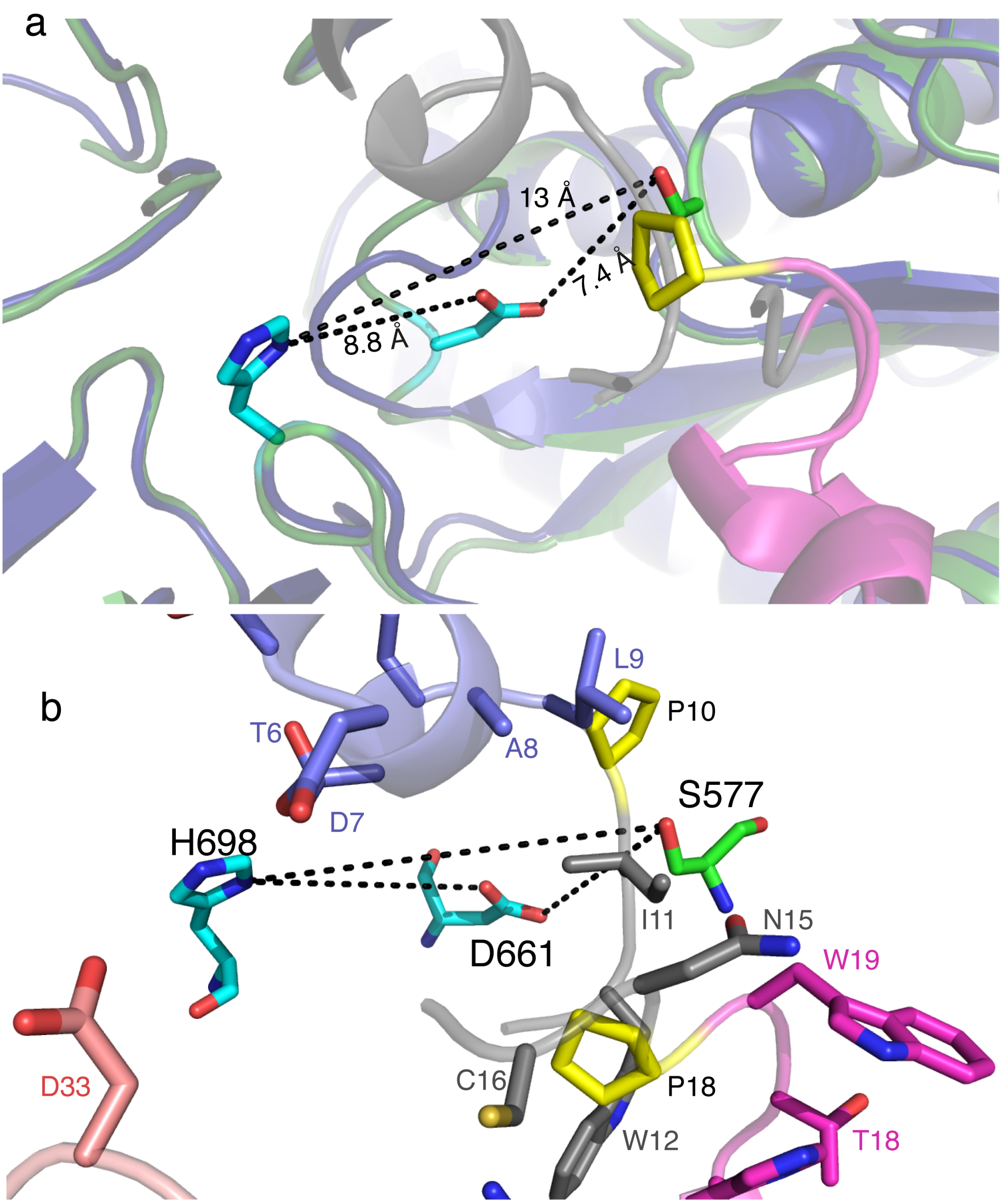

Supplementary Figure 7: Catalytic triad positions in the complex structures determined. **(a)** structures of D661A-25mer (blue) and S577A-35mer (green) are shown in cartoon, active site distances are shown. **(b)** 35mer peptide residues are shown in sticks, color coded as Figure 1b.

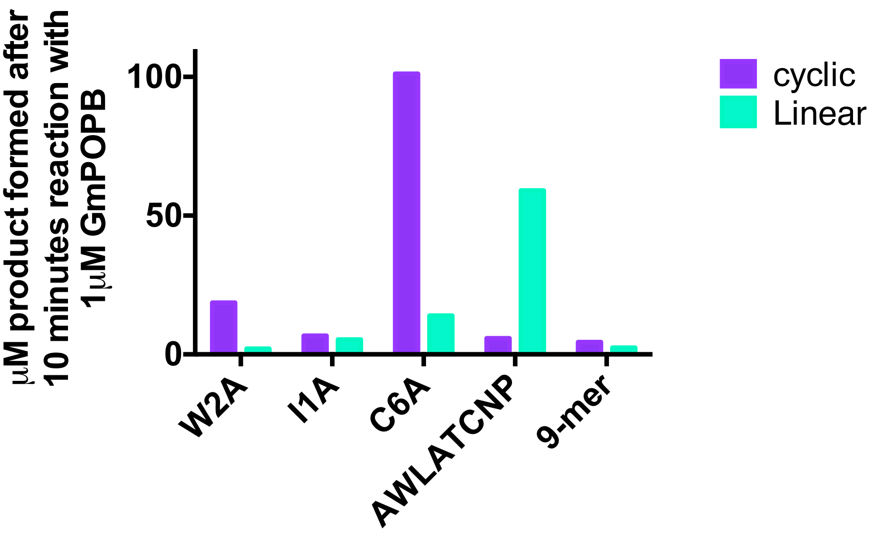

Supplementary Figure 8: Linear and cyclic peptide products when substrates with altered core sequences were used. LC-MS quantification using masses for the linear peptide produced by hydrolysis (859.3 Da) or cyclic peptide produced by macrocyclisation (841.3 Da). Data was quantified using a calibration curve with standards for linear and cyclic peptides.

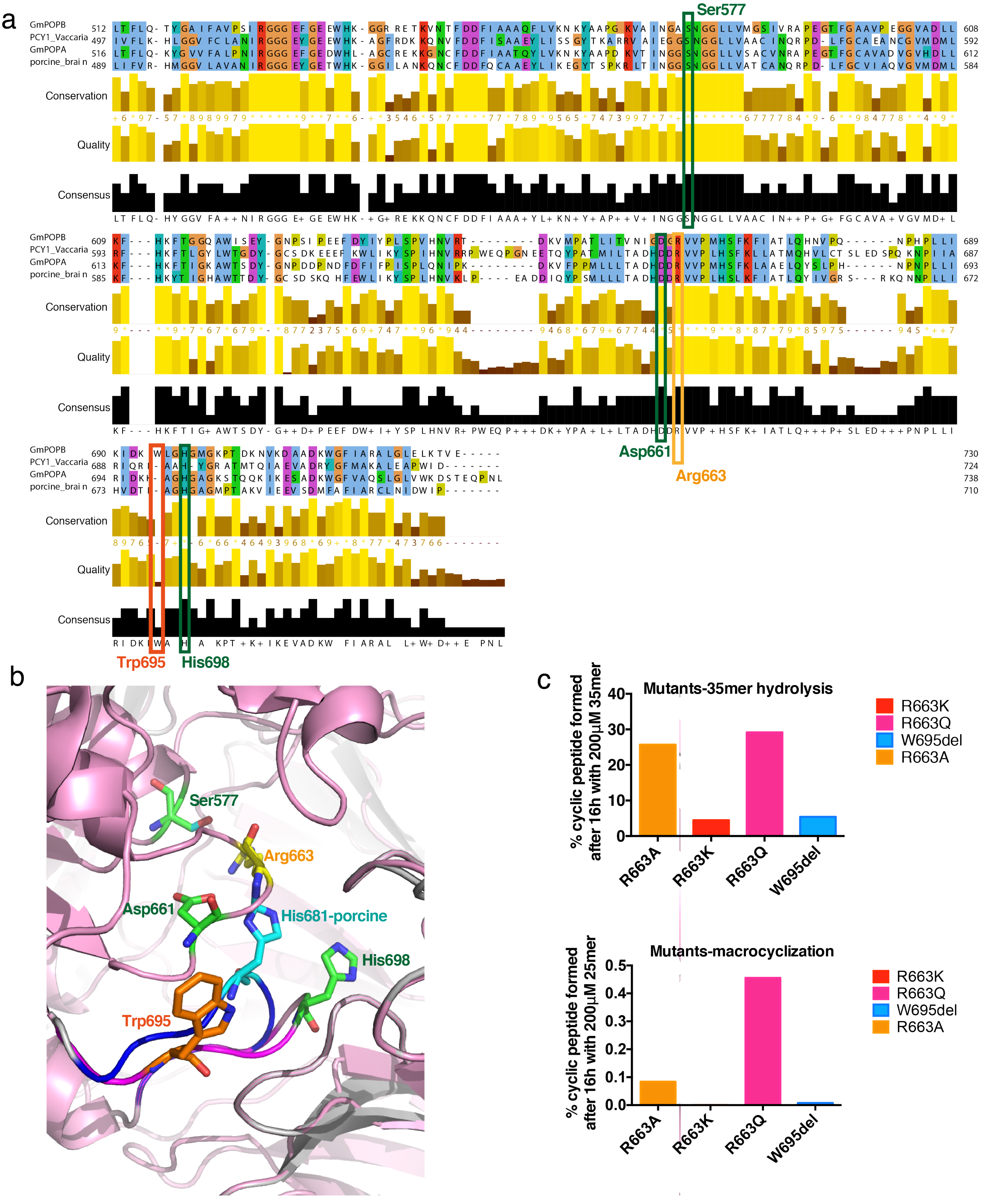

Supplementary Figure 9: Effect of mutations on peptide bond hydrolysis and macrocyclization. (a) Sequence alignment between GmPOPB, PCY1, GmPOPA and porcine brain POP. Alignment was performed using ClustalΩ and Jalview.[1, 2] Catalytic triad is shown in green, and residues mutated are shown as in panel b. (b) Position of residues mutated and catalytic triad. Porcine POPB catalytic His (His681) is shown for comparison. (c) Top: Reaction with the 35mer peptide, bars correspond to linear 25mer product formed after 16h reaction with 1µM each GmPOPB mutant. Under these conditions, negligible cyclic peptide formation was observed when the 35mer was used as substrate. Bottom: Macrocyclization reaction with the 25mer peptide. Bars correspond to cyclic peptide product formed after 16h reaction with 1µM each GmPOPB mutant. Data was quantified as described in materials and methods.

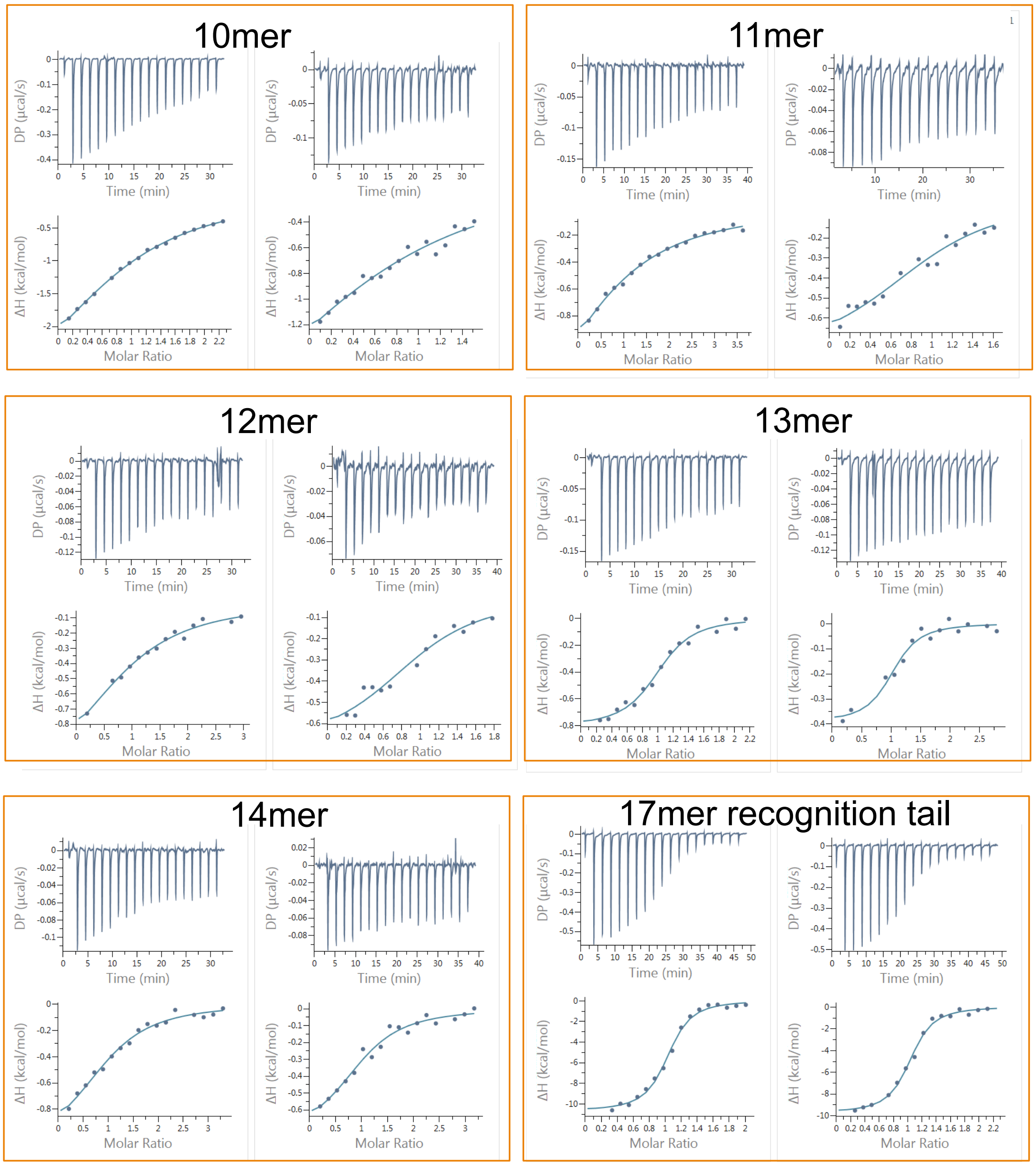

10mer
11mer
12mer
13mer
14mer
17mer recognition tail
DP (µcal/s)
Time (min)
ΔH (kcal/mol)
Molar Ratio

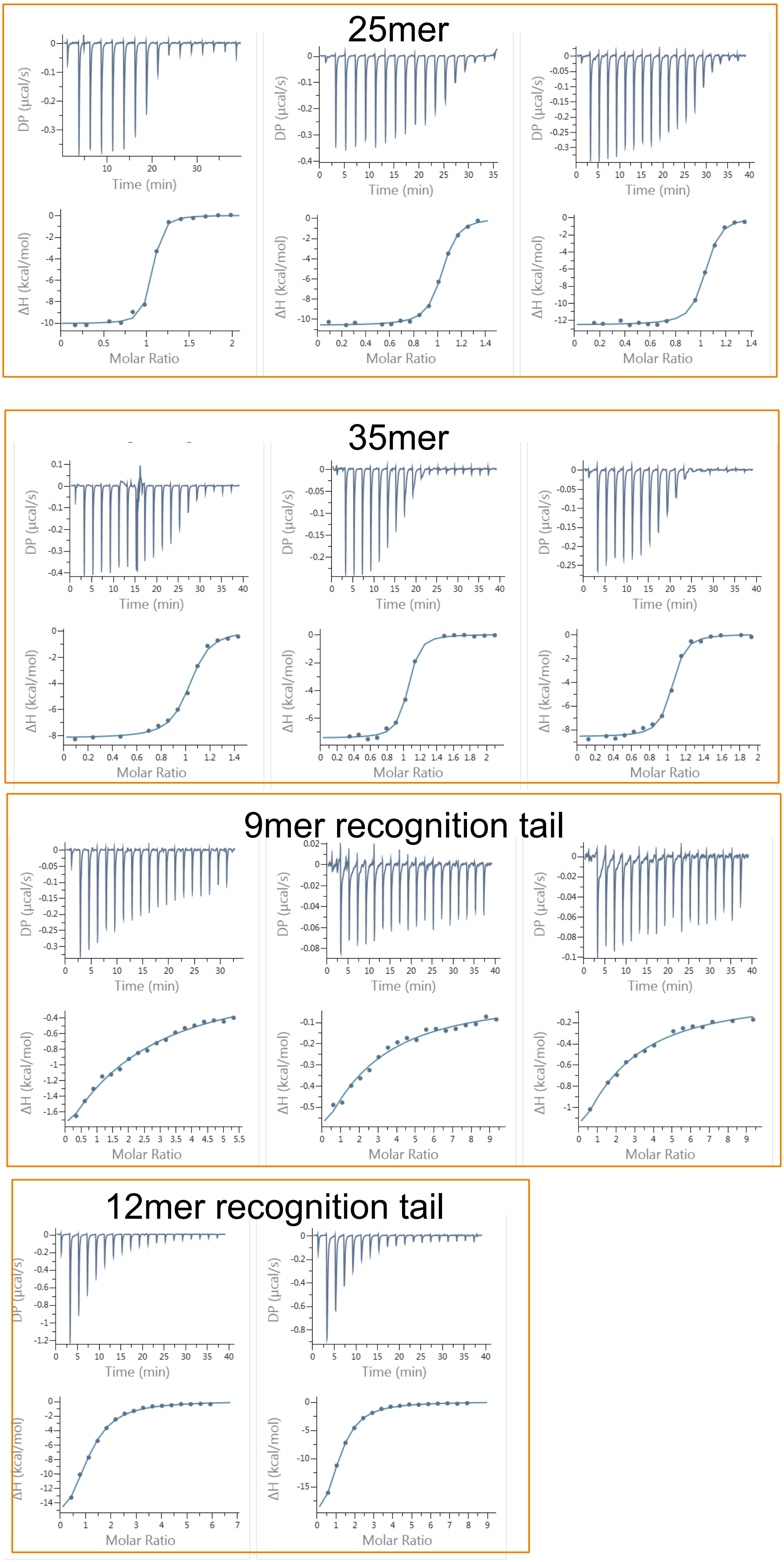

Supplementary Figure 10 ITC replicates for all peptides tested.

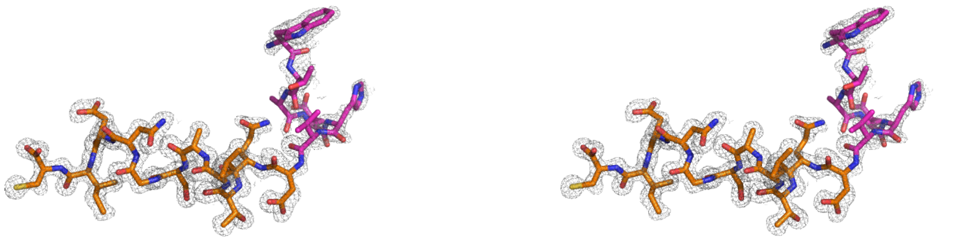

Stereo figure of the 25mer peptide bound to GmPOPB-S577A showing electron density for peptide ($2F_o - F_c$ contoured at 1 σ level in grey mesh).

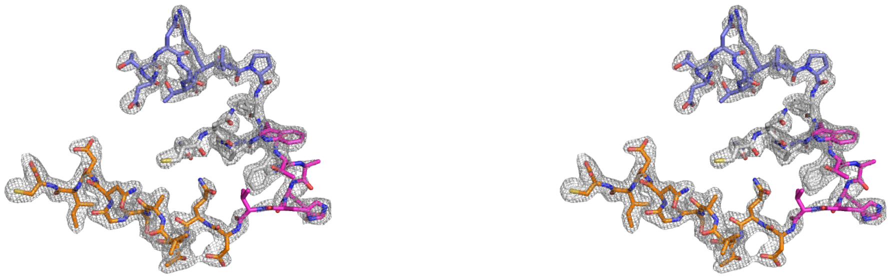

Stereo figure of the 35mer peptide bound to GmPOPB-S577A showing electron density ($2F_o - F_c$ contoured at 1 σ level in grey mesh) for peptide.

Supplementary Figure 11 Stereo figures of peptide ligand.

Supplementary Table 1 Primers used

**Primer name**	**Sequence (5’to 3’)**
**S577A-Forward**	**TGGTGCGGCCAACGGCGGCCTGCTGGTGATGGGC**
**S577A-Reverse**	**GCCGTTGGCCGCACCATTAATTGCAACTTTACCCGGAG**
**R663A-Forward**	**TGATGGCGCGGTGGTTCCGATGCACAGCTTCAAGTTCATCGC**
**R663A-Reverse**	**GAACCACCGCGCCATCACCGATATTCACGGTAATCAGGGTTGCC**
**R663Q-Forward**	**TGATGGCCAGGTGGTTCCGATGCACAGCTTCAAGTTCATCGC**
**R663Q-Reverse**	**GAACCACCTGGCCATCACCGATATTCACGGTAATCAGGGTTGCC**
**R663K-Forward**	**TGATGGCAAAGTGGTTCCGATGCACAGCTTCAAGTTCATCGC**
**R663K-Reverse**	**GAACCACTTTGCCATCACCGATATTCACGGTAATCAGGGTTGCC**
**D661A-Forward**	**TATCGGTGCGGGCCGTGTGGTTCCGATGCACAGCTTCAAGTTCATC**
**D661A-Reverse**	**ACGGCCCGCACCGATATTCACGGTAATCAGGGTTGCCGG**
**H698A-Forward**	**GTTGGGTGCCGGCATGGGTAAGCCGACCGACAAAAATGTGAAAG**
**H698A-Reverse**	**CATGCCGGCACCCAACCACGATTTATCAATCTTAATCAGCAGCGGAT[illegible]**
**H698N-Forward**	**GTTGGGTAACGGCATGGGTAAGCCGACCGACAAAAATGTGAAAG**
**H698N-Reverse**	**CATGCCGTTACCCAACCACGATTTATCAATCTTAATCAGCAGCGGATG**
**W695deletion-Forward**	**GATTGATAAATCGTTGGGTCACGGCATGGGTAAGCCGACCGAC**
**W695deletion-Reverse**	**CCATGCCGTGACCCAACGATTTATCAATCTTAATCAGCAGCGGATGC**
**GmPOPB_internalSequencing**	**CGTATTCGTTAACAAC**
**GmPOPB_T7Promoter**	**GAATGGCATAAGGGCGG**
**T7_Terminator**	**TAATACGACTCACTATAGG**
	**AAACCCCTCAAGACC**

Supplementary Table 2 Kinetic parameters for peptide substrates.

Substrate	k_{cat} (min^{-1})	K_m (μM)	k_{cat}/K_m ($\mu M^{-1}\ min^{-1}$)
**13mer**	0.49 ± 0.03	24.4 ± 5.2	0.020 ± 0.004
**14mer**	0.58 ± 0.10	380 ± 140	0.0010 ± 0.0001
**25mer**	18 ± 5	51 ± 18	0.35 ± 0.16
**35mer-protease**	3.2 ± 0.4	23 ± 11	0.14 ± 0.07
**AbAMA1**	35 ± 5	25 ± 13	1.36 ± 0.69
**I1L**	4.4 ± 0.6	19 ± 10	0.23 ± 0.12
**W2F**	4.4 ± 0.6	8 ± 5	0.52 ± 0.32
**C6S**	31 ± 6	28 ± 19	1.13 ± 0.80
**C25S**	8.8 ± 0.9	10 ± 5	0.9 ± 0.4

Supplementary Table 3 Thermodynamic parameters obtained by ITC.

Peptide	Sequence	K_d (µM)	ΔG (kcal mol^{-1})	ΔH (kcal mol^{-1})	-TΔS (kcal mol^{-1})	N measurements
**9mer**	IWGIGCNPW	ND	ND	ND	ND	
**10mer**	IWGIGCNPWT	83 ± 17	-5.5 ± 0.1	-3.1 ± 0.5	-2.4 ± 0.3	2
**11mer**	IWGIGCNPWTA	39 ± 18	-5.9 ± 0.3	-1.5 ± 0.7	4.5 ± 0.9	2
**12mer**	IWGIGCNPWTAE	21 ± 5	-6.3 ± 0.1	-1.0 ± 0.3	-5.3 ± 0.4	2
**13mer**	IWGIGCNPWTAEH	2.4 ± 0.1	-7.3 ± 0.1	-0.6 ± 0.2	-6.9 ± 0.2	2
**14mer**	IWGIGCNPWTAEHV	9.5 ± 1.1	-6.7 ± 0.1	-0.9 ± 0.2	-5.8 ± 0.2	2
**9mer-recognition sequence**	TLASGNDIC	121 ± 19	-5.3 ± 0.1	-6.4 ± 1.6	2.6 ± 1.5	3
**12mer-recognition sequence**	VDQTLASGNDIC	5 ± 1	-7.1 ± 0.1	-23.5 ± 3.1	16.3 ± 3.0	2
**17mer-recognition sequence**	WTAEHVDQTLASGNDIC	0.43 ± 0.01	-8.5 ± 0.1	-10.2 ± 0.4	1.7 ± 0.4	2
**25mer**		0.067 ± 0.014	-9.7 ± 0.1	-11.1 ± 0.7	1.4 ± 0.8	3
**35mer**		0.12 ± 0.03	-9.3 ± 0.1	-8.1 ± 0.0.3	-1.2 ± 0.4	3

ND – peptide solubility was lower than 300 µM and measurements could not be performed.

Errors reported are standard error of the mean using the replicates indicated in the table and the fitted values using Malvern data analysis software.

Supplementary references

[1] Waterhouse, A. M., Procter, J. B., Martin, D. M., Clamp, M., and Barton, G. J. (2009) Jalview Version 2--a multiple sequence alignment editor and analysis workbench, *Bioinformatics 25*, 1189-1191.

[2] Sievers, F., Wilm, A., Dineen, D., Gibson, T. J., Karplus, K., Li, W. Z., Lopez, R., McWilliam, H., Remmert, M., Soding, J., Thompson, J. D., and Higgins, D. G. (2011) Fast, scalable generation of high-quality protein multiple sequence alignments using Clustal Omega, *Molecular Systems Biology 7*.
